# Long-Term Disease Control With Lenvatinib in Metastatic Malignant Struma Ovarii: A Case Report

**DOI:** 10.7759/cureus.95507

**Published:** 2025-10-27

**Authors:** Ryota Kawano, Tomoko Sumikura, Kohki Shimazu, Masahiko Takemura, Atsuhiko Uno

**Affiliations:** 1 Otolaryngology - Head and Neck Surgery, Osaka General Medical Center, Osaka, JPN; 2 Obstetrics and Gynecology, Osaka General Medical Center, Osaka, JPN; 3 Pathology, Osaka General Medical Center, Osaka, JPN

**Keywords:** malignant struma ovarii, ovarian tumor, poorly differentiated thyroid carcinoma, thyroglobulin, tyrosine kinase inhibitor

## Abstract

Struma ovarii is a rare monodermal teratoma composed predominantly of thyroid tissue. Although most cases are benign, some are diagnosed as malignant on the basis of histological features, local invasion, or metastatic disease. We report a patient with malignant struma ovarii who has been maintained on long-term lenvatinib (LVB) therapy for postoperative metastatic disease. A woman in her 40s was incidentally found to have a pelvic mass after a fall. Resection of the ovarian tumor led to a diagnosis of struma ovarii, with small foci of thyroid-like tissue and predominantly areas resembling poorly differentiated thyroid carcinoma. Genomic profiling revealed an NRAS mutation. Because of residual disease, total thyroidectomy followed by radioactive iodine therapy was performed, but no uptake was observed. Subsequently, multiple hepatic metastases and pelvic dissemination developed, and conventional chemotherapy including bevacizumab was ineffective. LVB induced marked regression of hepatic and pelvic lesions. However, reductions in dose due to anorexia, fatigue, and proteinuria were accompanied by increased serum thyroglobulin (Tg) levels and progression of hepatic metastases. Switching to sorafenib led to further progression, but reintroduction of LVB reduced Tg levels. Treatment was interrupted for six months due to myocardial infarction and generalized peritonitis, during which Tg levels rose sharply and hepatic metastases regrew. After resuming LVB with dose reduction and planned drug holidays, disease stabilization was achieved, and the therapy has been maintained. The patient has survived seven years since recurrence, including six years on LVB. The tumor behaved similarly to poorly differentiated thyroid carcinoma, with Tg levels reflecting disease activity and LVB demonstrating the potential for long-term tumor control.

## Introduction

Struma ovarii is a monodermal teratoma composed predominantly or entirely of thyroid tissue. Although it is the most common type of monodermal teratoma, it remains rare, accounting for less than 3% of all ovarian tumors [1]. The majority of cases are benign, and only 0.1% to 5% are considered malignant, usually on the basis of postoperative recurrence or metastasis [2,3].

We report here the clinical course of a patient with malignant struma ovarii who underwent ovarian tumor resection, total thyroidectomy, and radioactive iodine therapy, and later developed metastatic disease that has been maintained on long-term lenvatinib (LVB) treatment. Reports on the use of tyrosine kinase inhibitors in malignant struma ovarii are extremely limited [3], making this case a valuable contribution to the literature.

## Case presentation

**Figure 1:**
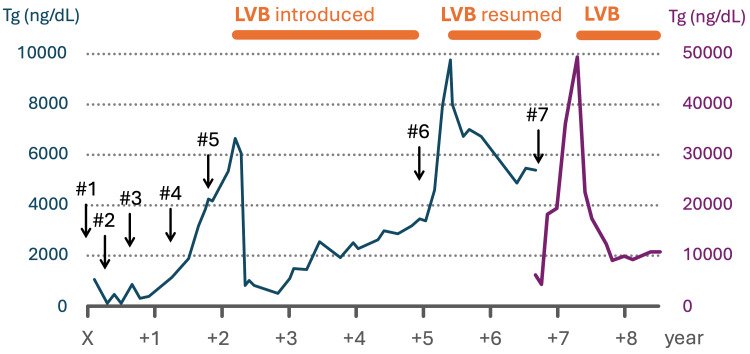
Changes in serum thyroglobulin (Tg) levels over time The X-axis represents the number of years since the ovarian tumor resection, and the Y-axis represents Tg levels (note the change in vertical scale in the latter part of the graph). Periods of lenvatinib (LVB) administration are indicated by horizontal bars above the graph. Clinical events are marked as follows: #1, ovarian tumor resection; #2, total thyroidectomy and initiation of thyrotropin-stimulating hormone (TSH) suppression; #3, radioactive iodine therapy; #4, progression of pelvic and hepatic metastases; #5, chemotherapy with paclitaxel, carboplatin, and bevacizumab (PTX/CBDCA/Bev); #6, temporary switch from LVB to sorafenib; #7, LVB interruption due to acute myocardial infarction and generalized peritonitis. The pace of tumor progression was clearly suppressed during LVB treatment.

**Figure 2:**
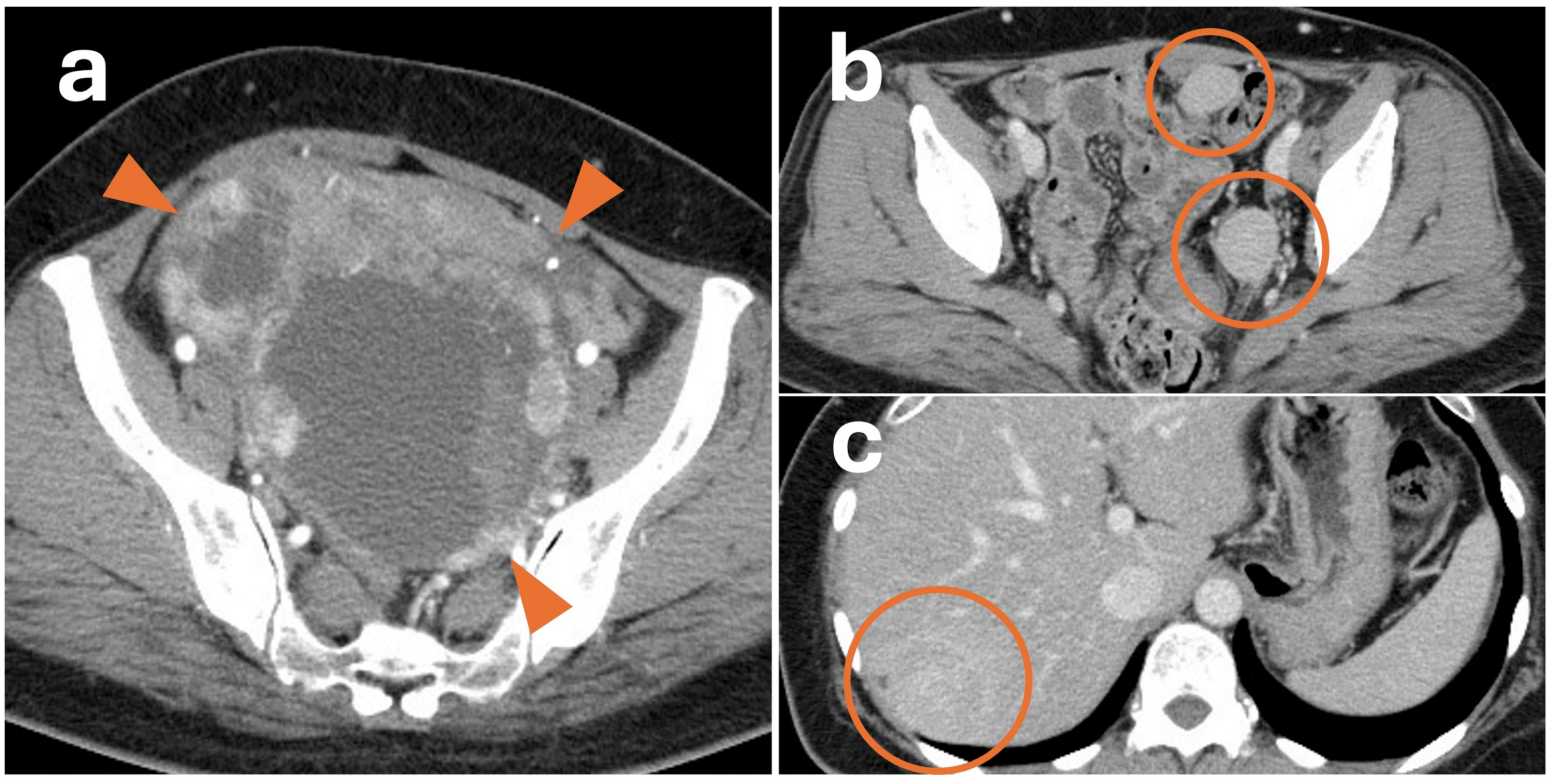
CT images of the tumor (a) Ovarian tumor occupying the pelvic cavity; (b) After surgery, enlargement of pelvic and (c) hepatic metastases was observed in association with rising thyroglobulin (Tg) levels (see also event #4 in Figure 1).

A woman in her 40s was incidentally found to have a pelvic mass on an MRI performed after a fall that resulted in a lumbar contusion. At initial presentation, she had comorbid type 2 diabetes mellitus (HbA1c, 9.4%) and hypertension. With a preoperative diagnosis of suspected right ovarian cancer, she underwent total hysterectomy with bilateral salpingo-oophorectomy and partial omentectomy (Figure 1, #1; Figure 2a).

**Figure 3:**
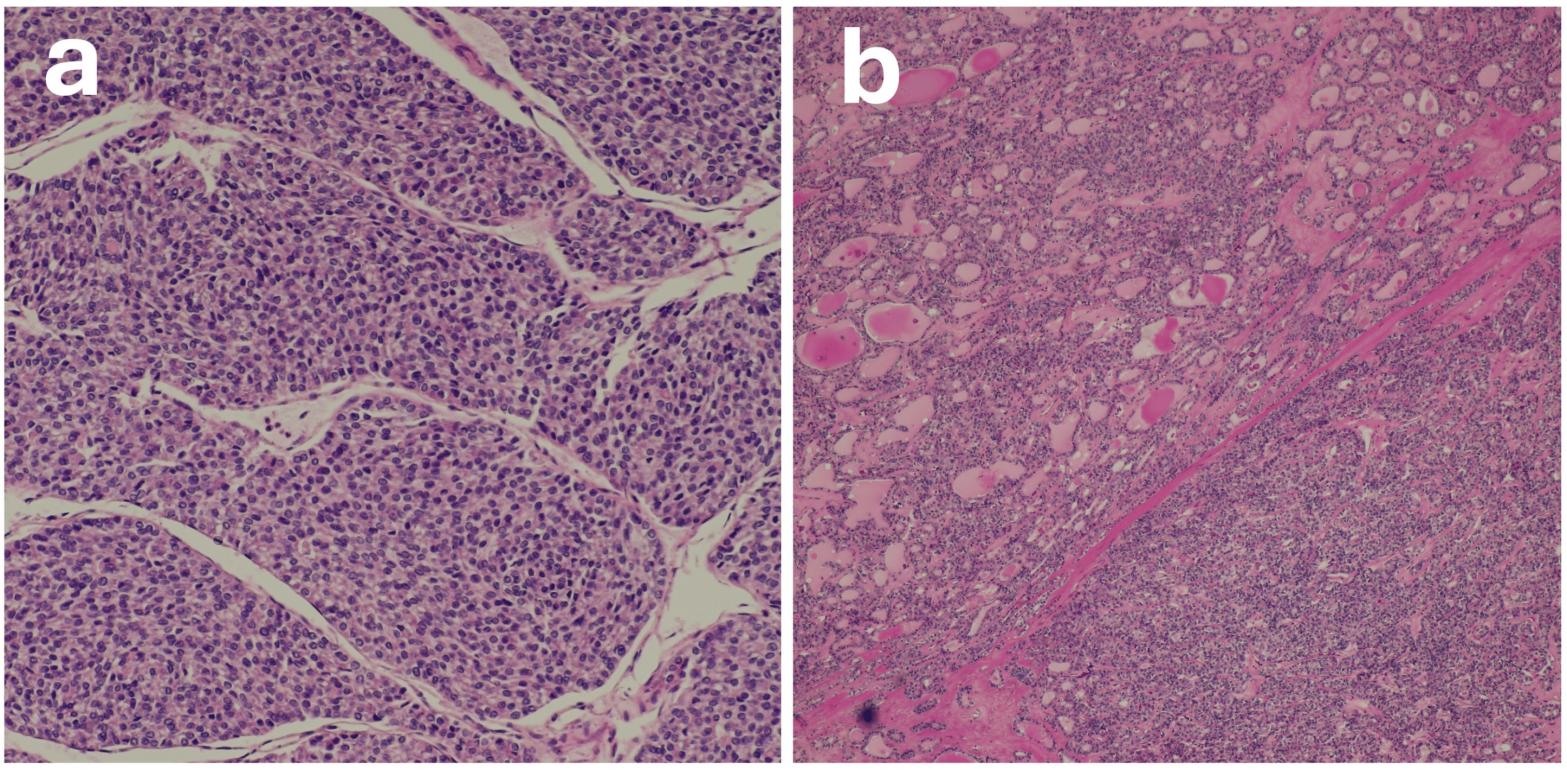
Pathological findings of the ovarian tumor (a) In most areas of the tumor, the neoplastic tissue shows trabecular and solid growth patterns; (b) At the tumor margin, there are areas where tumor cells of uniform size, resembling thyroid follicular epithelium, form follicular or tubular structures. For details, including immunohistochemical and genetic findings, see the main text.

Gross examination of the resected specimen revealed a right ovarian tumor measuring 20 cm in the greatest dimension. On the cut surface, the lesion was mostly gray-white and solid, with central areas of necrosis suggestive of ischemic change. A small portion at the periphery appeared yellowish-brown. Histopathological examination showed that the gray-white areas corresponded to regions exhibiting trabecular or solid growth patterns (Figure 3a).

Nucleoli were indistinct, and intranuclear inclusions or grooves were not apparent. Coagulative necrosis was limited to a small area, and mitotic figures were generally scarce, with a maximum density of two to three per 10 high-power fields. The yellowish-brown peripheral areas consisted of uniform-sized tumor cells resembling thyroid follicular epithelium, forming follicular and tubular structures (Figure 3b). Similar trabecular and solid tumor components, identical to those in the gray-white regions of the ovarian lesion, were also observed in the retroperitoneal lesion, showing expansile growth.

Immunohistochemistry demonstrated positivity for thyroid transcription factor-1 (TTF-1), cytokeratin 7 (CK7), thyroglobulin (Tg), cytokeratin 19 (CK19), Hector Battifora Mesothelial-1 (HBME-1), and epithelial (E)-cadherin; negativity for galectin-3 (GAL3), calcitonin, inhibin, synaptophysin, chromogranin A, Cluster of Differentiation 56 (CD56), Epithelial Membrane Antigen (EMA), tumor protein 53 (p53), and nuclear beta-catenin (with positive membranous staining); and focal/weak positivity for B-cell lymphoma 2 (Bcl-2). Based on these findings, the tumor was diagnosed as struma ovarii. Considering it as a malignant ovarian tumor and applying the classification of thyroid carcinoma, no nuclear features of papillary carcinoma were observed, and the tumor was regarded as corresponding to a poorly differentiated carcinoma arising from follicular carcinoma (see Discussion). Tumor genome profiling revealed microsatellite stability, a low tumor mutation burden, and the presence of an NRAS Q51R mutation.

Following total thyroidectomy, radioactive iodine therapy at a dose of 150 mCi was administered for residual local disease; however, no definite uptake was observed in the tumor, and the resected thyroid showed no remarkable pathological findings. Thyrotropin-stimulating hormone (TSH) suppressive therapy with slightly excessive doses of levothyroxine resulted in a corresponding decrease in serum Tg levels. Tg levels closely reflected disease activity, and rising Tg levels were accompanied by the detection of multiple hepatic metastases and pelvic dissemination (Figure 1, #2-4). Chemotherapy with paclitaxel, carboplatin, and bevacizumab (PTX/CBDCA/Bev) was administered, but the lesions progressed (Figure 1, #5).

Initiation of LVB at a starting dose of 24 mg daily led to marked shrinkage of hepatic and pelvic lesions. However, dose reductions and intermittent dosing schedules, such as 14-18 mg daily for nine days on and five days off, or 10 days on and four days off, were later required because of anorexia, fatigue, and proteinuria, and these adjustments were followed by rising Tg levels and enlargement of hepatic metastases. A temporary switch to sorafenib was attempted, but due to rapid disease progression, LVB was resumed, resulting in a subsequent decrease in Tg levels.

The patient later developed acute myocardial infarction and generalized peritonitis secondary to duodenal perforation (Figure 1, #7). A six-month interruption of LVB led to regrowth of hepatic metastases, an increase in Tg to over 50,000 ng/dL, and the appearance of anti-Tg antibodies. LVB was restarted at 14 mg/day on a five days on and two days off schedule, resulting in disease stabilization. This regimen has since been maintained.

## Discussion

The pathological findings of the ovarian tumor in this case, including the presence of thyroid follicle-like structures and positive immunohistochemical staining for Tg and TTF-1, supported a diagnosis of struma ovarii. However, even in areas showing extensive tumor proliferation, histology alone did not allow a definitive distinction between benign and malignant disease. Because struma ovarii resembles thyroid tissue, diagnostic criteria for thyroid tumors, such as nuclear features of papillary carcinoma or capsular and vascular invasion in follicular carcinoma, have been considered. Nevertheless, a distinct capsule or vascular structures are not always present within the ovary, making it difficult to apply the same invasion-based criteria used for thyroid tumors. Moreover, some cases of struma ovarii with bland cytology have shown recurrence or metastasis, whereas others with marked atypia have remained localized and clinically indolent [2,4]. Thus, the diagnosis of malignant struma ovarii ultimately depends on whether the tumor demonstrates clinically malignant behavior, such as recurrence or metastasis. In the present case, malignancy was suspected based on adhesive extension into adjacent organs, but it was confirmed only after the development of disseminated pelvic disease and multiple hepatic metastases.

For malignant or suspected malignant struma ovarii, total thyroidectomy followed by radioactive iodine therapy, analogous to the management of differentiated thyroid carcinoma, has been reported as effective [5], and this approach was undertaken in our patient. Although multiple recurrences were detected a few months after therapy, suggesting that radioactive iodine had little effect on the tumor, thyroidectomy and radioiodine ablation eliminated the need to consider normal thyroid tissue when interpreting rising serum Tg levels. Consequently, increases in Tg levels could be attributed to malignant struma ovarii. Indeed, subsequent changes in Tg closely reflected disease activity, which was corroborated by imaging studies. Because the patient developed postoperative hypothyroidism, levothyroxine replacement was required. When the levothyroxine dose was reduced, the resulting rise in TSH was accompanied by an increase in serum Tg levels. This behavior was consistent with that of thyroid follicular cell-derived tumors, and therefore TSH suppression therapy was undertaken in an attempt to inhibit tumor growth.

When reconsidered in light of the malignant clinical course, the histology of this tumor was consistent with poorly differentiated thyroid carcinoma. Solid and trabecular growth patterns, focal coagulative necrosis, and up to three mitotic figures per high-power field in the most active areas were observed, all features compatible with poorly differentiated carcinoma [6]. The presence of a NRAS mutation on tumor genomic profiling further supports the interpretation that this tumor represented a poorly differentiated carcinoma arising from follicular thyroid carcinoma.

The tumor in this case was considered indistinguishable from thyroid carcinoma, and after confirming that standard chemotherapy for ovarian carcinoma had little effect, LVB was introduced. LVB inhibits multiple receptor tyrosine kinases, including vascular endothelial growth factor receptors (VEGFRs), fibroblast growth factor receptors (FGFRs), and platelet-derived growth factor receptors (PDGFRs), and is established as the first-line therapy for radioactive iodine-refractory, recurrent, or metastatic thyroid carcinoma [7]. In our patient, LVB produced marked tumor suppression; however, adverse events such as anorexia, fatigue, and proteinuria were problematic. During the clinical course, myocardial infarction also occurred, which may have been related not only to preexisting diabetes mellitus and hypertension but also to LVB treatment.

The initial full dose could not be maintained, and dose reductions or temporary interruptions were required. Nevertheless, as shown in Figure 1, the steep increases in Tg levels observed prior to the introduction of LVB, during the period when sorafenib was administered instead, and during the interruption following myocardial infarction and peritonitis were clearly mitigated, though not completely suppressed, by LVB therapy. As a result, the treatment has been successfully continued for approximately seven years after the detection of recurrent and metastatic disease. The current regimen, consisting of LVB administration with planned drug holidays, has become a standard strategy to reduce toxicity while enabling long-term continuation of therapy [8].

The NRAS Q51R mutation observed in this case has been reported in pancreatic, colorectal, thyroid, and lung cancers, and is a statistically significant hotspot. However, no molecularly targeted therapy is currently available for this alteration. Therapeutic strategies aimed at NRAS itself and its downstream proliferative signaling pathways are under active development [9,10]. In this patient, continued survival under the current treatment may provide the opportunity to benefit from such novel therapies as they become available.

## Conclusions

We reported the clinical course of a patient diagnosed with malignant struma ovarii based on recurrence with metastasis. The tumor harbored a NRAS mutation and exhibited histological features consistent with poorly differentiated thyroid carcinoma, while serum Tg levels closely reflected tumor status. Although radioactive iodine therapy after total thyroidectomy was ineffective, rapid rises in Tg levels were suppressed by LVB. Periods of LVB discontinuation due to adverse events and myocardial infarction were associated with marked increases in Tg levels. Over approximately seven years since the detection of metastatic recurrence, the patient has survived with about six years of LVB treatment. It is hoped that future advances in novel therapeutic agents will provide treatment options applicable to this patient.

## References

[1] WHO Classification of Tumours Editorial Board (2025). Female Genital Tumours. WHO Classification of Tumours, 5th Edition, Volume 4. Female Genital Tumours. WHO Classification of Tumours, 5th Edition, Volume 4.

[2] Shaco-Levy R, Peng RY, Snyder MJ (2012). Malignant struma ovarii: a blinded study of 86 cases assessing which histologic features correlate with aggressive clinical behavior. Arch Pathol Lab Med.

[3] Taha T, Abu-Sini H, Billan S (2022). Tyrosine kinase inhibitor treatment and long-term follow-up for metastatic malignant struma ovarii. Pediatr Hematol Oncol.

[4] Garg K, Soslow RA, Rivera M, Tuttle MR, Ghossein RA (2009). Histologically bland "extremely well differentiated" thyroid carcinomas arising in struma ovarii can recur and metastasize. Int J Gynecol Pathol.

[5] Ayhan S, Kilic F, Ersak B (2021). Malignant struma ovarii: from case to analysis. J Obstet Gynaecol Res.

[6] WHO Classification of Tumours Editorial Board (2025). Endocrine and Neuroendocrine Tumours. WHO Classification of Tumours, 5th Edition, Volume 10. Endocrine and Neuroendocrine Tumours. WHO Classification of Tumours, 5th Edition, Volume 10.

[7] Ringel MD, Sosa JA, Baloch Z (2025). 2025 American Thyroid Association management guidelines for adult patients with differentiated thyroid cancer. Thyroid.

[8] Matsuyama C, Enokida T, Ueda Y (2023). Planned drug holidays during treatment with lenvatinib for radioiodine-refractory differentiated thyroid cancer: a retrospective study. Front Oncol.

[9] Wasiak K, Ciunowicz D, Kierasińska-Kałka A, Węgierska M, Pacholczyk M, Rieske P, Stoczyńska-Fidelus E (2025). The complex journey of targeting RAS in oncology. BMC Cancer.

[10] Hamidi S, Maniakas A, Akhave NS (2025). Characterization of advanced RAS-driven follicular-derived thyroid cancers and review of future therapeutic avenues. J Clin Endocrinol Metab.

